# 3,3′-{Ethane-1,2-diylbis[carbonylbis(azanediyl)]}dipyridinium tetra­chloridoplatinate(II)

**DOI:** 10.1107/S1600536810004253

**Published:** 2010-02-06

**Authors:** N. N. Adarsh, D. Krishna Kumar, Parthasarathi Dastidar

**Affiliations:** a Department of Organic Chemistry, Indian Association for the Cultivation of Science, 2A & 2B Raja S C Mullick Road, Jadavpur, Kolkata 700 032, India; b Present address: Department of Chemistry, Imperial College, London SW7 2AZ, England

## Abstract

In the crystal structure of the title compound, (C_14_H_18_N_6_O_2_)·[PtCl_4_], the cation and square-planar anion are located on special positions (on a twofold axis and an inversion centre, respectively). In the crystal structure, N—H⋯Cl hydrogen bonds lead to a staircase-like motif. The central ethane backbone of the cation is disordered over two positions of equal occupancy.

## Related literature

For organic–inorganic hybrid compounds displaying N—H⋯Cl—metal hydrogen bonds, see: Adams *et al.* (2007 ▶); Deifela & Cahill (2009 ▶). Orpen *et al.* (2004 ▶); Krishna Kumar *et al.* (2005 ▶, 2006 ▶).
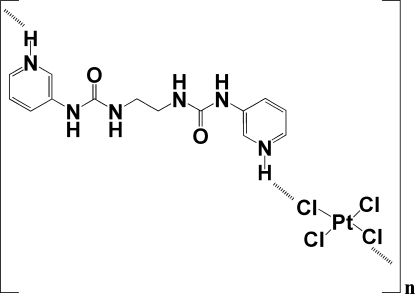


## Experimental

### 

#### Crystal data

(C_14_H_18_N_6_O_2_)[PtCl_4_]
*M* *_r_* = 639.23
Monoclinic, 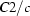

*a* = 17.8126 (13) Å
*b* = 7.0799 (5) Å
*c* = 15.5765 (12) Å
β = 103.332 (1)°
*V* = 1911.4 (2) Å^3^
*Z* = 4
Mo *K*α radiation
μ = 7.92 mm^−1^
*T* = 100 K
0.22 × 0.14 × 0.05 mm

#### Data collection

Bruker APEXII CCD area-detector diffractometer
Absorption correction: multi-scan (*SADABS*; Bruker, 2006 ▶) *T*_min_ = 0.275, *T*_max_ = 0.693
7045 measured reflections
1877 independent reflections
1693 reflections with *I* > 2σ(*I*)
*R*_int_ = 0.044

#### Refinement

*R*[*F* ^2^ > 2σ(*F* ^2^)] = 0.025
*wR*(*F* ^2^) = 0.064
*S* = 0.97
1877 reflections
132 parameters
H atoms treated by a mixture of independent and constrained refinement
Δρ_max_ = 1.51 e Å^−3^
Δρ_min_ = −0.89 e Å^−3^

### 

Data collection: *APEX2* (Bruker, 2006 ▶); cell refinement: *APEX2* and *SAINT* (Bruker, 2006 ▶); data reduction: *SAINT*; program(s) used to solve structure: *SHELXS97* (Sheldrick, 2008 ▶); program(s) used to refine structure: *SHELXL97* (Sheldrick, 2008 ▶); molecular graphics: *Mercury* (Macrae *et al.*, 2006 ▶) and *ORTEP-3* (Farrugia, 1997 ▶); software used to prepare material for publication: *SHELXL97*, *publCIF* (Westrip, 2010 ▶) and *PLATON* (Spek, 2009 ▶).

## Supplementary Material

Crystal structure: contains datablocks I, global. DOI: 10.1107/S1600536810004253/bt5171sup1.cif

Structure factors: contains datablocks I. DOI: 10.1107/S1600536810004253/bt5171Isup2.hkl

Additional supplementary materials:  crystallographic information; 3D view; checkCIF report

## Figures and Tables

**Table 1:** Hydrogen-bond geometry (Å, °)

*D*—H⋯*A*	*D*—H	H⋯*A*	*D*⋯*A*	*D*—H⋯*A*
N1—H1⋯Cl2^i^	0.76 (5)	2.55 (5)	3.259 (4)	156 (5)

Symmetry code: (i) 
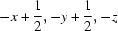
.

**Fig. 1.**
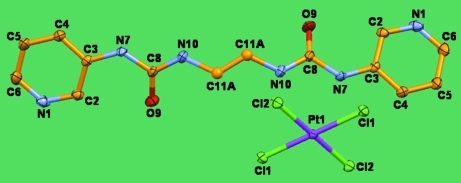
Ortep diagram of title compound with anisotropic displacement ellipsoids drawn at the 50% probability level. H atoms omitted for clarity.

**Fig. 2.**
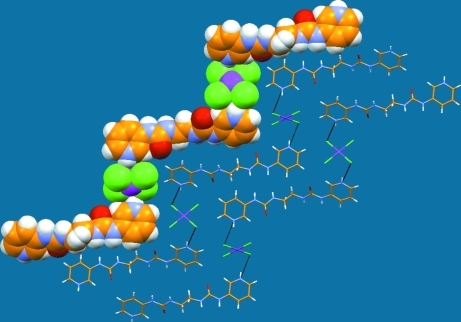
Staircaise like hydrogen bonded network via N—H···Cl interactions

## supplementary crystallographic information

### Comment

Suitable single crystals of the title compound were obtained by the resultant reaction of BPEBU and [K_2_(PtCl_4_)] in acidic medium (see experimental). The asymmetric unit consists of half of (PtCl_4_) and one half molecule of bis-protonated BPEBU. The ORTEP diagram with 50% probability is given in Fig. 1. The metal center Pt(II) displayed a slight distorted square plannar geometry [Cl—Pt—Cl = 89.91 (4)–90.09 (4)°]. In the crystal structure, the bis-protonated ligand BPEBU displays *syn–anti-syn* conformation (scheme 1). N—H···Cl hydrogen bonds lead to a staircase like structure (Fig. 2).

### Experimental

Synthesis: The title compound was synthesized by layering a methanolic solution of BPEBU (30 mg, 0.1 mmol) containing HCl (MeOH : HCl = 8 ml : 2 ml) over an aqueous solution of [K_2_(PtCl_4_)] (41.5 mg, 0.1 mmol) containings HCl (H_2_O : HCl = 8 ml : 2 ml). After a period of two weeks, X-ray quality single crystals were obtained (yield = 30 mg, 47%). FT–IR (KBr, cm-1): 3325 (b, O—H, N—H stretch), 3082 (b, aromatic C—H stretch), 2965 (b, aliphatic C—H), 1686 (s, urea C═O stretch), 1612 (s, urea N—H bend), 1580, 1520, 1480, 1422, 1331, 1264,1238, 1191, 1111, 1024, 989, 864, 800, 694.

### Refinement

H-atoms bonded to C were refined using a riding model with C—H ranging from 0.95 Å to 0.99 Å and U(H) = 1.2U_eq_(C). The coordinates of the H atoms bonded to N were refined with U(H) = 1.2U_eq_(N). The central C—C bond in the cation (located on the 2 fold axis) is disordered over two equally occupied positions.

### Crystal data

**Table:**

(C_14_H_18_N_6_O_2_)[PtCl_4_]	*F*(000) = 1224
*M**_r_* = 639.23	*D*_x_ = 2.221 Mg m^−^^3^
Monoclinic, *C*2/*c*	Mo *K*α radiation, λ = 0.71073 Å
*a* = 17.8126 (13) Å	Cell parameters from 566 reflections
*b* = 7.0799 (5) Å	θ = 2.4–27.8°
*c* = 15.5765 (12) Å	µ = 7.92 mm^−^^1^
β = 103.332 (1)°	*T* = 100 K
*V* = 1911.4 (2) Å^3^	Plate, yellow
*Z* = 4	0.22 × 0.14 × 0.05 mm

### Data collection

**Table:**

Bruker APEXII CCD area-detector diffractometer	1877 independent reflections
Radiation source: fine-focus sealed tube	1693 reflections with *I* > 2σ(*I*)
graphite	*R*_int_ = 0.044
φ–ω scan	θ_max_ = 26.0°, θ_min_ = 2.4°
Absorption correction: multi-scan (*SADABS*; Bruker, 2006)	*h* = −21→21
*T*_min_ = 0.275, *T*_max_ = 0.693	*k* = −8→8
7045 measured reflections	*l* = −19→19

### Refinement

**Table:**

Refinement on *F*^2^	Primary atom site location: structure-invariant direct methods
Least-squares matrix: full	Secondary atom site location: difference Fourier map
*R*[*F*^2^ > 2σ(*F*^2^)] = 0.025	Hydrogen site location: inferred from neighbouring sites
*wR*(*F*^2^) = 0.064	H atoms treated by a mixture of independent and constrained refinement
*S* = 0.97	*w* = 1/[σ^2^(*F*_o_^2^) + (0.0343*P*)^2^ + 6.3133*P*] where *P* = (*F*_o_^2^ + 2*F*_c_^2^)/3
1877 reflections	(Δ/σ)_max_ < 0.001
132 parameters	Δρ_max_ = 1.51 e Å^−^^3^
0 restraints	Δρ_min_ = −0.89 e Å^−^^3^

### Special details

**Table:**

Geometry. All esds (except the esd in the dihedral angle between two l.s. planes) are estimated using the full covariance matrix. The cell esds are taken into account individually in the estimation of esds in distances, angles and torsion angles; correlations between esds in cell parameters are only used when they are defined by crystal symmetry. An approximate (isotropic) treatment of cell esds is used for estimating esds involving l.s. planes.
Refinement. Refinement of *F*^2^ against ALL reflections. The weighted *R*-factor wR and goodness of fit *S* are based on *F*^2^, conventional *R*-factors *R* are based on *F*, with *F* set to zero for negative *F*^2^. The threshold expression of *F*^2^ > σ(*F*^2^) is used only for calculating *R*-factors(gt) etc. and is not relevant to the choice of reflections for refinement. *R*-factors based on *F*^2^ are statistically about twice as large as those based on *F*, and *R*- factors based on ALL data will be even larger.

### Fractional atomic coordinates and isotropic or equivalent isotropic displacement parameters (Å^2^)

**Table:**

	*x*	*y*	*z*	*U*_iso_*/*U*_eq_	Occ. (<1)
Pt1	0.5000	0.5000	0.0000	0.01584 (10)	
Cl1	0.39171 (5)	0.65021 (15)	−0.07921 (6)	0.0228 (2)	
Cl2	0.50224 (6)	0.69911 (14)	0.11747 (7)	0.0226 (2)	
N1	0.1742 (2)	−0.0639 (6)	−0.1087 (3)	0.0250 (8)	
H1	0.139 (3)	−0.108 (7)	−0.096 (3)	0.030*	
C2	0.2331 (3)	−0.0133 (5)	−0.0434 (3)	0.0193 (9)	
H2	0.2297	−0.0254	0.0163	0.023*	
C3	0.3001 (2)	0.0577 (6)	−0.0639 (2)	0.0160 (8)	
C4	0.3007 (2)	0.0717 (7)	−0.1528 (3)	0.0223 (8)	
H4	0.3455	0.1183	−0.1690	0.027*	
C5	0.2379 (3)	0.0195 (6)	−0.2176 (3)	0.0305 (12)	
H5	0.2391	0.0310	−0.2781	0.037*	
C6	0.1730 (3)	−0.0497 (7)	−0.1941 (3)	0.0304 (10)	
H6	0.1288	−0.0861	−0.2378	0.036*	
N7	0.36384 (19)	0.1145 (5)	−0.0011 (2)	0.0176 (7)	
C8	0.3757 (2)	0.0918 (6)	0.0896 (3)	0.0214 (9)	
O9	0.3290 (2)	0.0132 (4)	0.1240 (3)	0.0367 (10)	
N10	0.4417 (2)	0.1661 (6)	0.1358 (2)	0.0275 (9)	
C11A	0.4643 (5)	0.2153 (13)	0.2283 (5)	0.0202 (16)*	0.50
H11A	0.4227	0.1819	0.2582	0.024*	0.50
H11B	0.4740	0.3528	0.2351	0.024*	0.50
C11B	0.4631 (5)	0.1063 (12)	0.2315 (5)	0.0185 (16)*	0.50
H11C	0.4728	−0.0314	0.2365	0.022*	0.50
H11D	0.4213	0.1381	0.2614	0.022*	0.50
H7	0.400 (3)	0.164 (6)	−0.020 (3)	0.022*	
H10	0.467 (3)	0.219 (7)	0.116 (3)	0.032*	

### Atomic displacement parameters (Å^2^)

**Table:**

	*U*^11^	*U*^22^	*U*^33^	*U*^12^	*U*^13^	*U*^23^
Pt1	0.01344 (14)	0.01733 (15)	0.01748 (14)	−0.00161 (7)	0.00507 (9)	−0.00225 (7)
Cl1	0.0172 (5)	0.0302 (5)	0.0211 (5)	0.0044 (4)	0.0046 (4)	0.0037 (4)
Cl2	0.0192 (5)	0.0254 (5)	0.0239 (5)	−0.0025 (4)	0.0062 (4)	−0.0096 (4)
N1	0.0220 (19)	0.0246 (19)	0.030 (2)	−0.0071 (16)	0.0105 (16)	−0.0072 (17)
C2	0.019 (2)	0.019 (2)	0.021 (2)	−0.0005 (14)	0.0066 (19)	−0.0024 (15)
C3	0.020 (2)	0.0134 (17)	0.0147 (19)	0.0009 (16)	0.0045 (16)	−0.0006 (16)
C4	0.020 (2)	0.030 (2)	0.018 (2)	−0.0010 (18)	0.0067 (17)	−0.0012 (18)
C5	0.023 (3)	0.050 (3)	0.018 (2)	0.0020 (19)	0.004 (2)	−0.0089 (18)
C6	0.023 (2)	0.040 (2)	0.026 (3)	−0.002 (2)	0.003 (2)	−0.017 (2)
N7	0.0184 (17)	0.0221 (18)	0.0135 (16)	−0.0055 (13)	0.0064 (14)	0.0016 (13)
C8	0.019 (2)	0.033 (2)	0.0128 (19)	0.0068 (17)	0.0043 (16)	0.0044 (17)
O9	0.028 (2)	0.063 (3)	0.0219 (18)	−0.0011 (14)	0.0108 (16)	0.0151 (14)
N10	0.022 (2)	0.046 (2)	0.0143 (18)	−0.0013 (16)	0.0037 (15)	−0.0045 (16)

### Geometric parameters (Å, °)

**Table:**

Pt1—Cl1^i^	2.2971 (9)	C6—H6	0.9500
Pt1—Cl1	2.2971 (9)	N7—C8	1.389 (5)
Pt1—Cl2^i^	2.3028 (9)	N7—H7	0.83 (5)
Pt1—Cl2	2.3028 (9)	C8—O9	1.223 (5)
N1—C6	1.330 (6)	C8—N10	1.337 (6)
N1—C2	1.331 (6)	N10—C11A	1.447 (8)
N1—H1	0.76 (5)	N10—C11B	1.511 (9)
C2—C3	1.397 (6)	N10—H10	0.71 (5)
C2—H2	0.9500	C11A—C11B^ii^	1.513 (10)
C3—N7	1.377 (5)	C11A—H11A	0.9900
C3—C4	1.392 (5)	C11A—H11B	0.9900
C4—C5	1.374 (7)	C11B—C11A^ii^	1.513 (10)
C4—H4	0.9500	C11B—H11C	0.9900
C5—C6	1.380 (7)	C11B—H11D	0.9900
C5—H5	0.9500		
			
Cl1^i^—Pt1—Cl1	180.0	C3—N7—C8	126.6 (3)
Cl1^i^—Pt1—Cl2^i^	90.11 (4)	C3—N7—H7	116 (3)
Cl1—Pt1—Cl2^i^	89.89 (4)	C8—N7—H7	117 (3)
Cl1^i^—Pt1—Cl2	89.89 (4)	O9—C8—N10	123.1 (4)
Cl1—Pt1—Cl2	90.11 (4)	O9—C8—N7	122.6 (4)
Cl2^i^—Pt1—Cl2	180.0	N10—C8—N7	114.3 (4)
C6—N1—C2	124.9 (4)	C8—N10—C11A	129.8 (5)
C6—N1—H1	118 (4)	C8—N10—C11B	114.3 (4)
C2—N1—H1	117 (4)	C11A—N10—C11B	30.3 (4)
N1—C2—C3	119.0 (4)	C8—N10—H10	122 (4)
N1—C2—H2	120.5	C11A—N10—H10	105 (4)
C3—C2—H2	120.5	C11B—N10—H10	123 (4)
N7—C3—C4	119.4 (4)	N10—C11A—C11B^ii^	107.6 (6)
N7—C3—C2	123.4 (4)	N10—C11A—H11A	110.2
C4—C3—C2	117.2 (4)	C11B^ii^—C11A—H11A	110.2
C5—C4—C3	121.3 (4)	N10—C11A—H11B	110.2
C5—C4—H4	119.3	C11B^ii^—C11A—H11B	110.2
C3—C4—H4	119.3	H11A—C11A—H11B	108.5
C4—C5—C6	119.4 (5)	N10—C11B—C11A^ii^	105.2 (5)
C4—C5—H5	120.3	N10—C11B—H11C	110.7
C6—C5—H5	120.3	C11A^ii^—C11B—H11C	110.7
N1—C6—C5	118.1 (4)	N10—C11B—H11D	110.7
N1—C6—H6	121.0	C11A^ii^—C11B—H11D	110.7
C5—C6—H6	121.0	H11C—C11B—H11D	108.8
			
C6—N1—C2—C3	−1.1 (7)	C3—N7—C8—O9	1.0 (7)
N1—C2—C3—N7	179.7 (4)	C3—N7—C8—N10	−177.7 (4)
N1—C2—C3—C4	0.2 (6)	O9—C8—N10—C11A	−17.3 (8)
N7—C3—C4—C5	−178.9 (4)	N7—C8—N10—C11A	161.5 (6)
C2—C3—C4—C5	0.6 (6)	O9—C8—N10—C11B	13.7 (7)
C3—C4—C5—C6	−0.5 (7)	N7—C8—N10—C11B	−167.5 (5)
C2—N1—C6—C5	1.2 (7)	C8—N10—C11A—C11B^ii^	122.2 (6)
C4—C5—C6—N1	−0.3 (7)	C11B—N10—C11A—C11B^ii^	53.4 (10)
C4—C3—N7—C8	−172.8 (4)	C8—N10—C11B—C11A^ii^	−176.5 (5)
C2—C3—N7—C8	7.7 (6)	C11A—N10—C11B—C11A^ii^	−48.3 (10)

Symmetry codes: (i) −*x*+1, −*y*+1, −*z*; (ii) −*x*+1, *y*, −*z*+1/2.

### Hydrogen-bond geometry (Å, °)

**Table:**

*D*—H···*A*	*D*—H	H···*A*	*D*···*A*	*D*—H···*A*
N1—H1···Cl2^iii^	0.76 (5)	2.55 (5)	3.259 (4)	156 (5)

Symmetry codes: (iii) −*x*+1/2, −*y*+1/2, −*z*.
